# Coffee Flower Identification Using Binarization Algorithm Based on Convolutional Neural Network for Digital Images

**DOI:** 10.34133/2020/6323965

**Published:** 2020-10-06

**Authors:** Pengliang Wei, Ting Jiang, Huaiyue Peng, Hongwei Jin, Han Sun, Dengfeng Chai, Jingfeng Huang

**Affiliations:** ^1^Institute of Applied Remote Sensing and Information Technology, Zhejiang University, Hangzhou 310058, China; ^2^Jiangsu Radio Scientific Institute Co., Ltd., Wuxi 214073, China; ^3^Innovation Institute of Disaster Prevention and Reduction at Inner Mongolia, Huhhot 010051, China; ^4^Institute of Spatial Information Technique, Zhejiang University, Hangzhou 310027, China; ^5^Electrical Engineering and Computer Science, University of California, Merced, CA 95343, USA

## Abstract

Crop-type identification is one of the most significant applications of agricultural remote sensing, and it is important for yield estimation prediction and field management. At present, crop identification using datasets from unmanned aerial vehicle (UAV) and satellite platforms have achieved state-of-the-art performances. However, accurate monitoring of small plants, such as the coffee flower, cannot be achieved using datasets from these platforms. With the development of time-lapse image acquisition technology based on ground-based remote sensing, a large number of small-scale plantation datasets with high spatial-temporal resolution are being generated, which can provide great opportunities for small target monitoring of a specific region. The main contribution of this paper is to combine the binarization algorithm based on OTSU and the convolutional neural network (CNN) model to improve coffee flower identification accuracy using the time-lapse images (i.e., digital images). A certain number of positive and negative samples are selected from the original digital images for the network model training. Then, the pretrained network model is initialized using the VGGNet and trained using the constructed training datasets. Based on the well-trained CNN model, the coffee flower is initially extracted, and its boundary information can be further optimized by using the extracted coffee flower result of the binarization algorithm. Based on the digital images with different depression angles and illumination conditions, the performance of the proposed method is investigated by comparison of the performances of support vector machine (SVM) and CNN model. Hence, the experimental results show that the proposed method has the ability to improve coffee flower classification accuracy. The results of the image with a 52.5° angle of depression under soft lighting conditions are the highest, and the corresponding Dice (F1) and intersection over union (IoU) have reached 0.80 and 0.67, respectively.

## 1. Introduction

Coffee is one of the top three major beverages worldwide and has important economic value. *Coffea arabica* L. is native to Africa and cultivated in several provinces in China, especially in Yunnan Province. Early coffee flower monitoring is of paramount importance in flowering regulation, irrigation, yield prediction, and other crop management tasks [1, 2]; therefore, the accurate identification of coffee flowers is the key to better managing these tasks. Nowadays, based on various data platforms, a number of datasets have been generated and developed for crop-type identification [3–8]. Generally, for different identification fields, the datasets can be divided into two main categories: datasets that are based on manual observation [9, 10] and datasets that are based on satellite platform observation [11–13]. However, as the demand for observation grows, there are several disadvantages for plant identification in small-scale plantations using these types of datasets, which are explained as follows.

While traditional manual observation is conducted by meteorologists on a regular basis [14], because of the limitation of subjectivity and the number of personnel, it is difficult to achieve objective and continuous observation for many years. Consequently, the traditional manual observations are mainly applied to small and medium scope and short-term observations. For instance, Ohashi et al. [9] studied the relationship between cherry blossom and climate, and in the process of cherry blossom identification, the flowering observation dates were quite fixed and only for a specific number of cherry trees; thus, it was difficult to efficiently obtain precise data. In addition, there have been many crowdsourcing projects that use manual observation in plant phenology monitoring, such as observation of apple flowering in Germany [6], cherry blossom flowering period in Japan [10], the Project BudBurst in the United States [15], and so on [16]. However, it is difficult to measure the human cost in these methods, and there are subjective limitations and data irregularities in the traditional manual observation, which cause difficultly in applying this type of data into low-density and high altitude areas.

Compared with manual observation, it is more convenient to obtain datasets from satellite platforms, and the datasets can be used to conduct continuous observation at different scales for many years [17, 18]. Remote sensing technologies based on satellite platforms have been well developed for vegetation observation [19–22], and they also have been applied to coffee phenology observation. For instance, MODIS datasets with high temporal resolution and coarse spatial resolution have been applied to observe coffee plantations in Brazil, which were aimed at predicting the distribution of coffee plants and observing their phenology characteristics [23]. Similarly, Couto et al. [24] used the MODIS data to extract the time series change of the normalized vegetation index and enhanced vegetation index, which could be used to analyze the differences between coffee and other land covers. However, it is difficult to achieve high spatial and temporal resolution simultaneously in such experiments, since the satellite platform is mostly based on large scale. Moreover, the precise positioning of small targets (i.e., coffee flowers) cannot be achieved because of the coarser resolution of data from satellite platforms. In recent years, the applications of unmanned aerial vehicles (UAVs) have been well expanded to agriculture, and they have already been used for small-scale observation [2]; the possibilities of continuous and long-term observation are limited, since the technical cost and the cost of labor of a single UAV flight are also high.

Some achievements have been made by using the above datasets; however, accurate small target monitoring still cannot be achieved in space and time. Consequently, in order to obtain the distribution of small targets in small-scale plantations, and provide a reliable theoretical basis for real-time monitoring, the use of high spatial-temporal resolution imaging is necessary. Fortunately, with the development of near-ground remote sensing technology, the emergence of time-lapse images that have high spatial-temporal resolution has effectively solved the problem of small target recognition and achieved the vegetation development monitoring in near real time for specific areas [25–31]. For instance, Zhang et al. [32] monitored crop growth using time-lapse images and calculated nine color vegetation indices from the acquired time series digital photographs to arrive at a fractional vegetation cover estimation model for different crops. Tan et al. [33] used simple linear iterative clustering (SLIC) for wheat spike identification in digital images based on the super green value and normalized red green index. Moreover, Peng et al. [34] have already proposed an SVM model based on superpixel merging (i.e., SPMG) to identify coffee flowers using digital images. Consequently, time-lapse images with high spatial-temporal resolution will play an increasingly important role in providing accurate and continuous data for small target monitoring.

Although a time-lapse image can play a better role in small-scale observation, its recognition accuracy needs to be further improved. The main reason it has low recognition accuracy is that the identification is based on traditional algorithms that only consider pixel-level features and ignore the space characteristics. With the development of computer vision technology, deep learning has attracted more and more attention in the field of image recognition, due to its ability to extract multiscale features, and it has been well migrated to the application of time-lapse image identification [35–37]. For instance, Xiong et al. [38] combined the SLIC and convolutional neural network (CNN) to identify rice panicles in digital images. Desai et al. [39] used CNN to detect regions containing flowering panicles from digital images of paddy rice, which can be used to estimate the heading date of the crop. However, for the traditional CNN models, in order to achieve image segmentation, a local region (patch) around a pixel, which is generated by using a sliding window, is selected to be the input of the CNN model to acquire the label of the center pixel of the patch. Mainly because of the existence of nontarget objects in the patch, the boundary of the target will be overrecognized in the final segmentation result, which is especially true in the case of small targets.

Therefore, in order to further improve coffee flower identification accuracy, based on time-lapse images, this paper utilizes the binarization algorithm and deep learning model to combine the pixel-level and space characteristics to discriminate coffee flowers from a complex background, and images with different depression angles and illumination conditions are identified using different models to validate the advantages of the combination of the binarization algorithm and deep learning model.

## 2. Materials and Methods

### 2.1. Study Area and Image Acquisition

**Figure 1:**
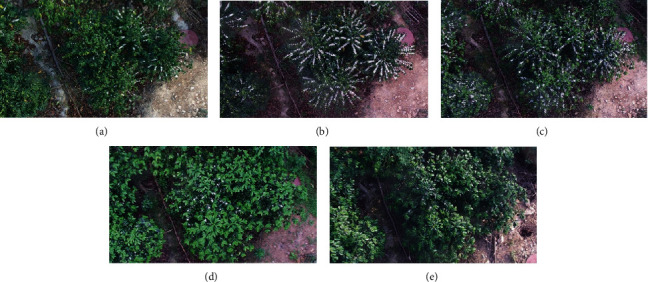
The five flowering events. (a–e) represent the images acquired on March 7^th^, March 25^th^, April 11^th^, April 27^th^, and May 25^th^, 2017, respectively.

The experimental field is a coffee plantation located in Lujiangba District of Baoshan, Yunnan Province, China. The annual average temperature is 21.3°C, the absolute maximum temperature is 40.4°C, and the absolute minimum temperature is 0.2°C. The climate of this area is categorized as a subtropical dry hot valley. The anthesis of the coffee in the study area occurs from March to May. The images used in the experiment were taken by an automatic observation device under natural illumination conditions. The device is mounted at an altitude of 5.8 m above ground level (Figure S1 (a)). A CCD image sensor is adapted by the camera, and its pan-and-tilt head cloud platform can be set to 24 different shooting angles, composed of 3 depression angles (i.e., 27.5°, 52.5°, and 77.5°) and 8 azimuth angles (i.e., 45° apart) (Figure S1 (b)). The resolution of the device is two million pixels, and the size of images acquired from the device is 1920(length) × 1080(width) pixels. The acquired digital images were shot at 08:00, 09:00, 10:00, 11:00, 12:00, 13:00, 14:00, 15:00, 16:00, 17:00, and 17:30 every day from March 1st to May 31st in 2017, during which five flowering events were observed (Figure 1).

**Figure 2:**
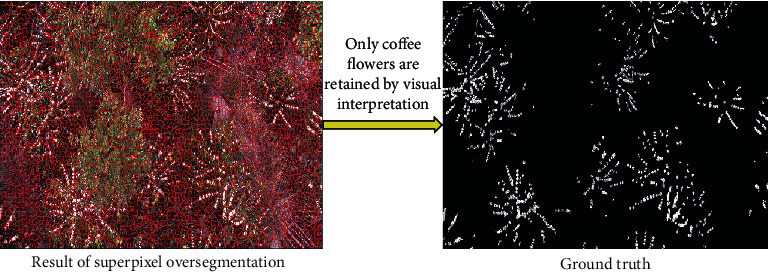
The process of generating ground truth.

In order to quantitatively analyze the identification accuracy of coffee flowers, the methods based on superpixel segmentation and visual interpretation are combined to build the ground truth map, the superpixel that contains the coffee flower is set to 1, and the background is set to 0. Then, the ground truth maps, under different illumination conditions and shooting depression angles, can be produced (Figure 2).

### 2.2. The Binarization Algorithm Based on CNN

CNN has been well applied to the field of image recognition. Generally, the structures of the CNN model mainly include the convolution layer, pooling layer, and full connection layer. The number of network parameters is reduced through weight sharing, which can improve the efficiency of the network. The pooling layer can ensure the invariance of displacement, scaling, and distortion while the feature dimension is reduced. For the setting of the network structure, the convolution layer and pooling layer are usually crossconfigured, and activation layers are set after each hidden layer (including the convolution layer and full connection layer) to achieve nonlinear transformation and accelerate the convergence speed of the network. Therefore, an arbitrarily complex CNN structure can be designed through different ways of configuring the convolution, pooling, and activation layers. However, there is still no effective theoretical guidance on how to design a CNN network so that it can meet the performance requirements. Experiments and even empirical intuition are still effective methods by which to design a CNN network with high performance. Currently, the LeNet [40], AlexNet [41], VGGNet [42], and GoogLeNet [43] are typical structures of a CNN model. Among them, VGGNet is a deep CNN network composed of multiple convolution layers with the size of 3 × 3. Moreover, the advantages of this network have been verified in the classification task of ImageNet, and there are many classification and detection networks that have been designed based on the structure of VGGNet [44, 45].

Consequently, VGGNet is selected as the basic network structure for coffee flower identification. Details of the specific network structure are located in [42]. The main difference is that there are only two channels in the last fully connected layer, since coffee flower identification is a two-category classification problem (Table 1). Each convolution layer is followed by a ReLU layer to speed up the network convergence. Starting from the input image, the features flow downwards through the left and right layers and finally reach the output layer (Table 1).

**Figure 3:**
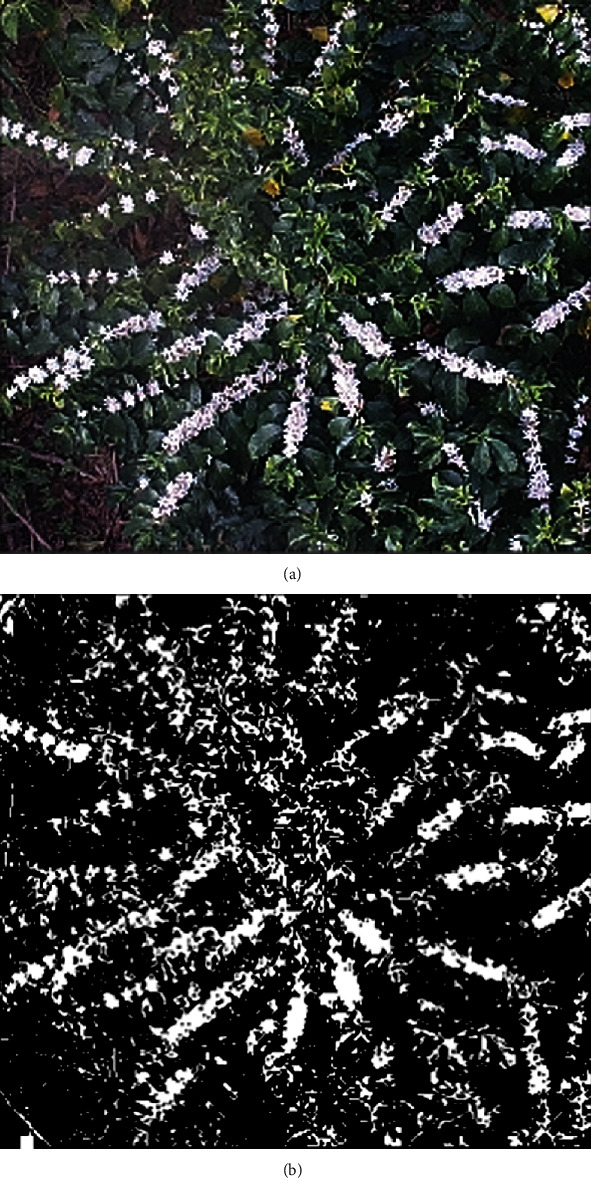
Binarization processing: (a) original image; (b) results of the binarization.

Through visual interpretation, it can be found that there are noticeable color differences between the coffee flower (white) and the surrounding background (Figure 3(a)), which means that an optimal threshold can be found to maximize the variance between the two classes to achieve the rough extraction of coffee flowers. Therefore, the binary transformation based on OTSU [46] is used to extract the contour information of the coffee flowers in the original image; from the segmentation result, it can be seen that the outlines of the coffee flowers can be effectively extracted by the binarization algorithm (i.e., OTSU) (Figure 3(b)).

**Figure 4:**
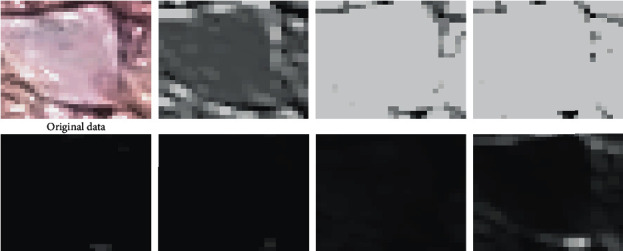
The extracted features of the background. The first image is the original input data; others are the extracted features from the third set of convolutional layers.

However, some background noise, which is relatively close to the pixel value of coffee flowers, has also been extracted and misclassified as coffee flowers, since the OTSU algorithm is a color-based threshold method. Moreover, there is a striking difference in the spatial information between the background and the coffee flower, and the difference can be extracted by CNN and used to improve this kind of misclassification that remains in the binarization result. In order to illustrate that the background information close to the color of the coffee flower can be suppressed by the CNN model, a brighter background image is classified, and the features extracted by the third convolution layer are visualized (Figure 4). It can be found from the visualization result that there are some solid color images in the extracted features, due to the highly consistent spatial features in the image block (i.e., all of the pixels in the image block are background information). At the same time, there are also some grayscale images similar to the original image, because of the influence caused by the different color information of the background in the image block. Consequently, both color and spatial information of the image block that correspond to the background can be learned by the trained CNN, which is the reason that the VGGNet can effectively suppress the excessive misclassification caused by the color value-based threshold method. However, there will be some redundant information for the coffee flower boundary in the path-based CNN results, mainly because the entire image block will be classified into the same category.

Therefore, in order to optimize coffee flower recognition accuracy, only the redundant background information that remains in the binarization result is removed using the results of path-based CNN, and the coffee flower information extracted by the binarization is still retained. The specific steps are described as follows:

**Step 1 (extraction of training datasets).**

A certain number of positive samples (i.e., coffee flowers) from the images with coffee flowers and negative samples (i.e., background) from the images without coffee flowers are extracted according to the specified neighborhood window size.

**Step 2 (training of CNN model).**

The initialized network model is trained using the extracted positive and negative samples.

**Step 3 (prediction).**

In forecasting the image comprised of prediction sets, the whole image is predicted using a sliding window based on the trained CNN model, so as to achieve the initial recognition of coffee flower and background information, and then the identification results are saved.

**Step 4 (extraction of boundary information).**

The binarization algorithm based on OTSU is used to find an optimal threshold to maximize the variance between the coffee flower and the background, which can effectively separate the boundary contour information of the coffee flower from the background.

**Step 5 (correction of boundary information).**

Finally, the boundary information of the coffee flowers acquired from Step 4 is used to limit the contour range of coffee flowers identified by Step 3, so as to achieve the optimization purpose of the patch-based CNN model. In other words, the result of the patch-based CNN model is used to remove the background information left in the binarization result.

Therefore, the CNN model and binarization algorithm are combined (Bin+CNN) to further optimize the coffee flower identification results.

**Figure 5:**
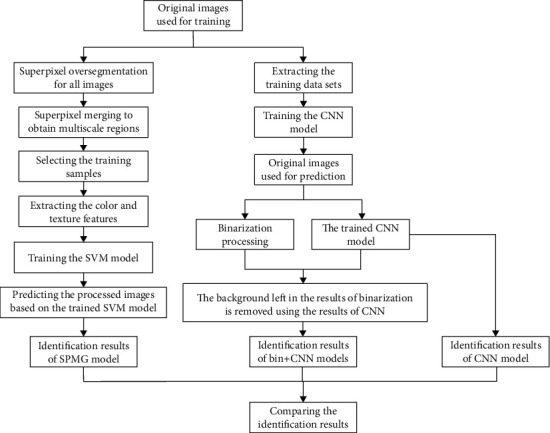
The flow chart of the research.

In order to validate the advantages of the proposed method (i.e., Bin+CNN), the coffee flower identification results based on the method presented in [34] (i.e., SPMG), CNN, and Bin+CNN models are compared (Figure 5).

### 2.3. Selection of the Training Datasets

The training samples, composed of positives (flower regions) and negatives (background regions), are selected from multiscale regions of images. Forty images are used to select positive samples, which were taken from 5 different flowering times, 11 shooting hours, and 9 different angles (i.e., 3 depression angles and 3 azimuth angles). Twelve images, which were taken from five flowering dates without flower images, are used to select negative samples.

**Figure 6:**
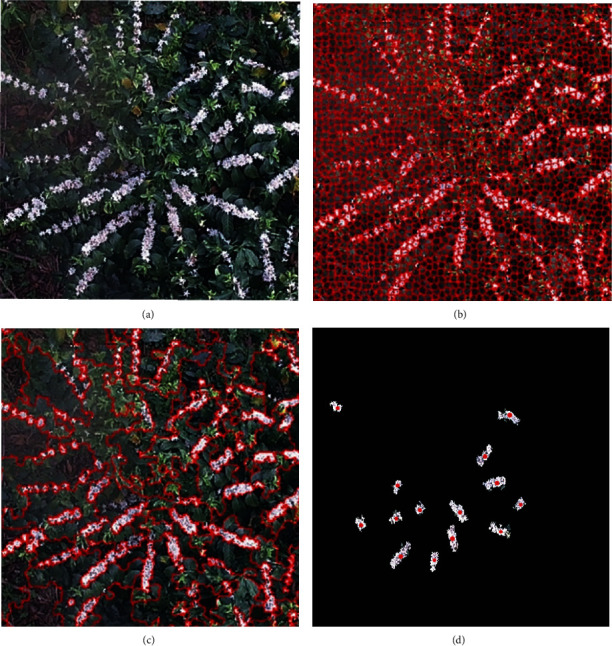
Generation of multiscale regions: (a) original image, (b) superpixel oversegmentation, (c) superpixel merging, and (d) the merged sample sets and coordinate point of the corresponding sample.

For the training datasets of the SVM model based on superpixel merging, the image is first divided into 15,000 superpixels by SLICO algorithm (i.e., SLIC zero) [47], and then 1500 merged regions can be obtained for each image (Figure 6). By combining similar regions and removing duplicates, the final merged sample sets can be obtained (Figure 6(c)), and there are a total of 3087 unequal coffee flower sample areas in all the images that contain coffee flowers. The center coordinates of all of the merged regions are recorded (i.e., red points in Figure 6(d)) to facilitate the positive sample selection of the CNN model. For negative samples, images without coffee flowers are selected. Compared with coffee flower information, the background information is more complicated, which means that more background information needs to be selected as negative samples. In the same way, superpixel segmentation and merging are carried out, respectively. One thousand five hundred merged regions were obtained for each image, and the final number of the negative sample sets is 18,000.

**Figure 7:**
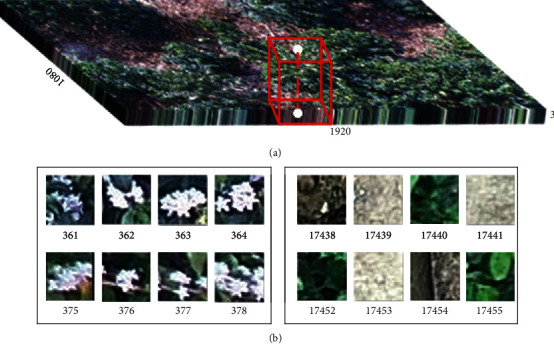
The input of the CNN model: (a) example of the input data based on the CNN model (i.e., the red image block with a size of 31 × 31 × 3); (b) parts of the positive and negative training samples.

For the training datasets of the CNN model, the coordinates of coffee flowers, which are recorded in the above selection process, are used as the center of the neighborhood block (i.e., white point in Figure 7(a)). Therefore, the center point of each image block (i.e., positive sample of CNN) corresponds to the center of each merged superpixel (i.e., positive sample of SVM) one by one. Mainly because the result extracted by CNN is used to remove the background information left in the binarization result, the window size should cover the complete coffee flower. Otherwise, the boundary information extracted by binarization will also be shrunk by CNN. Finally, the size of the image block is taken as 31 × 31, since the size of the coffee flower is about 900 pixels (Figure 7(b)). For negative samples, 1500 neighborhood blocks are randomly selected using the same way for each image.

### 2.4. CNN Training

**Figure 8:**
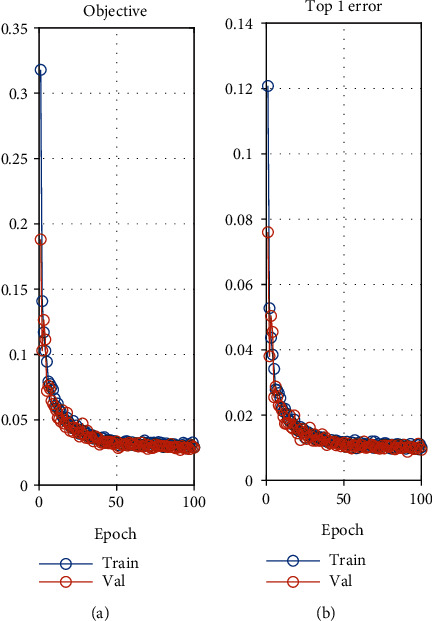
The training process of the CNN model: (a) the loss of all samples in a batch; (b) the loss of misidentification samples in a batch.

The specific parameter settings of network training are described as follows. For the SPMG model, the relevant parameters and kernel function are selected according to reference [34]. For the CNN model, a minibatch random gradient descent method with momentum factor is used to train the network, the number of each minibatch sample is set to 100, and the momentum factor is set to 0.9 [48], which is a fixed value. The weights of all of the layers are initialized using a Gaussian distribution with a mean of 0 and a standard deviation of 0.01 [42]. The biases of all of the convolutional layers and fully connected layers are initialized to 0. The initial learning rate is set to 0.01. When the accuracy error rate of the verification set is gradually stable during the training process, the training speed is slowed down by changing the learning rate to 10% of the current learning rate until the maximum training step is reached (i.e., 100). The specific process of the training loss is recorded (Figure 8). For the Bin+CNN model, the corresponding parameters of the network are set in the same way.

The modeling process is performed on a Windows workstation (Windows 10) with an Intel Xeon Gold 5218 Processor (16-core, 16M cache), 128 GB of RAM, and an NVIDIA Quadro P4000 graphics card (8 GB of RAM). Both of the deep learning models are implemented on the MATLAB platform using the MatConvNet library.

### 2.5. Assessment of the Identification Accuracy

In this study, the model performance is evaluated by comparing the recall, precision, Dice (F1), and the intersection over union (IoU), and the last two parameters are indicators that comprehensively consider recall and precision [34]. The corresponding parameters can be calculated using

(1) $$\begin{array}{c} \text{Recall}=\frac{\text{TP}}{\text{TP}+\text{FN}}, \end{array}$$

(2) $$\begin{array}{c} \text{Precision}=\frac{\text{TP}}{\text{TP}+\text{FP}}, \end{array}$$

(3) $$\begin{array}{c} \text{F}1=\frac{2\left|R_{\mathrm{seg}}\cap R_{\text{gt}}\right|}{\left|R_{\mathrm{seg}}\right|+\left|R_{\text{gt}}\right|}=\frac{2\text{TP}}{2\text{TP}+\text{FP}+\text{FN}}, \end{array}$$

(4) $$\begin{array}{c} \text{IoU}=\frac{\left|R_{\mathrm{seg}}\cap R_{\text{gt}}\right|}{\left|R_{\mathrm{seg}}\cup R_{\text{gt}}\right|}=\frac{\text{TP}}{\text{TP}+\text{FP}+\text{FN}}, \end{array}$$

where TP is the true positive, FP is the false positive, FN is the false negative, TN is the true negative, *R*_seg_ is the results of identification, and *R*_gt_ is the ground truth.

## 3. Results

The coffee flower images with different depression angles and illumination conditions are identified by SPMG, CNN, and Bin+CNN models, and the corresponding coffee flower identification results are compared to validate advantages of the proposed method (i.e., the Bin+CNN model).

### 3.1. Comparison Based on Different Methods for Training Datasets

**Figure 9:**
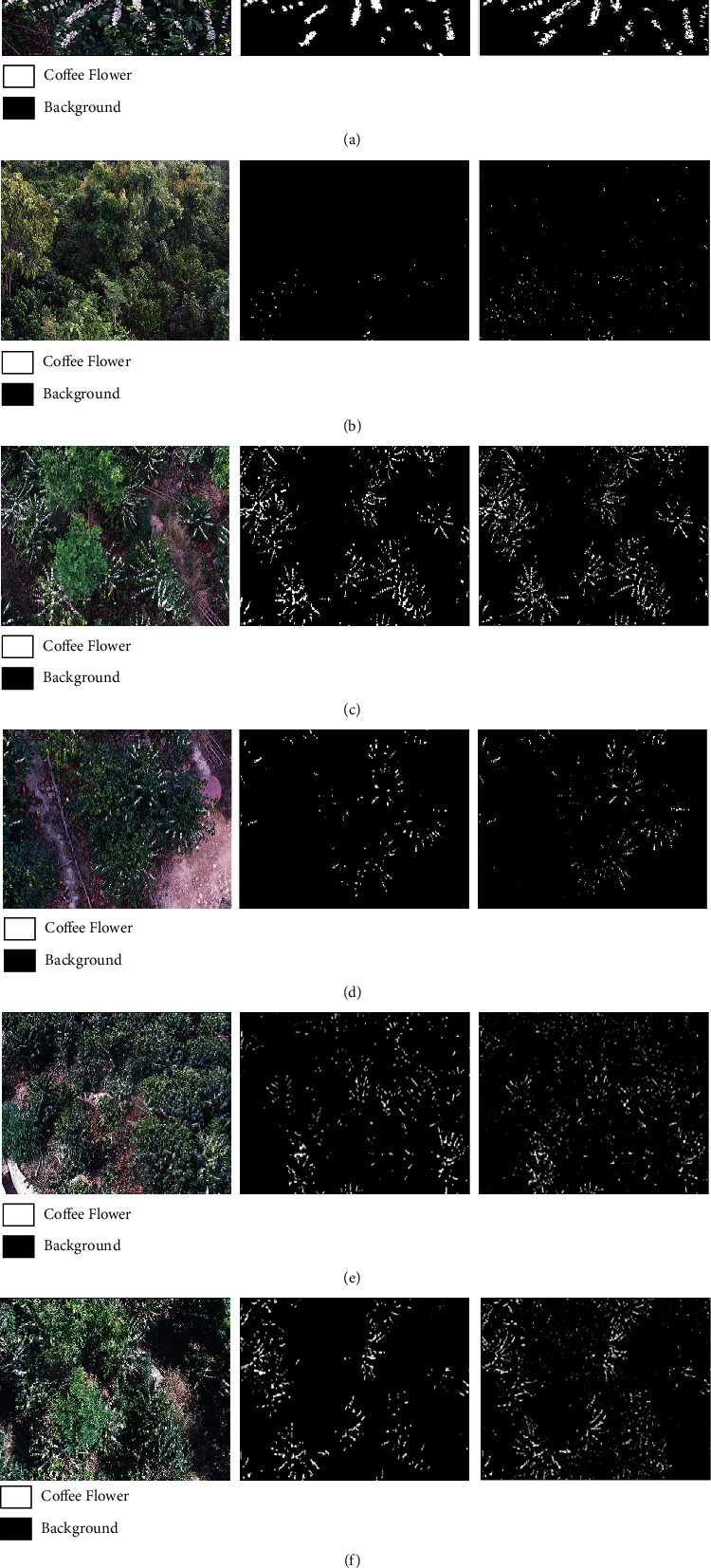
Coffee flower identification results of the Bin+CNN for different datasets. (a) Result of training data sets. (b–d) are the results of images with depression angles of 27.5°, 52.5°, and 77.5°under soft lighting conditions, respectively. (e, f) are the results of images with depression angles of 27.5° and 52.5°under intense lighting conditions, respectively. In addition, the first, middle, and third columns are the original images, ground truth maps, and identification results of the Bin+CNN, respectively.

One of the training images is selected to validate the trained SVM and CNN models. From the specific identification results (Figure 9(a), Figure S2, and Table 2), it can be seen that the recall rate of the CNN model reached 0.93, which is the highest compared to the other methods. However, there is too much false identification because the boundary information is amplified, which makes its other evaluation parameters low. For the results of the SPMG model, conversely, there is much more missing identification, since the spatial characteristics are ignored. For the results of the Bin+CNN model, as we expected, it can effectively correct the boundaries of the coffee flowers extracted by the CNN model, and the corresponding evaluation parameters have been clearly improved compared to those of the CNN models, and its recall, F1, and IoU are 0.16, 0.03, and 0.04 higher than those of the SPMG model, respectively.

### 3.2. Comparison Based on Different Methods and Depression Angles for Test Datasets

In order to further validate the robustness and advantages of the proposed method, the test images with different depression angles (i.e., 27.5°, 52.5°, and 77.5°) and illumination conditions (i.e., soft and intense lighting conditions) are identified using different methods, and the corresponding results are analyzed as follows.

#### 3.2.1. Results of the Image with a 27.5° Angle of Depression

The coffee flower image with a 27.5° angle of depression under soft lighting conditions is identified using the SPMG, CNN, and Bin+CNN models (Figure 9(b), Figure S3, and Table 3).

From the identification results, it can be seen that coffee flowers cannot be clearly displayed in the image with a 27.5° angle of depression, which makes it more difficult to recognize distribution information of coffee flowers. For the results of the SPMG model, the coffee flowers are hardly recognized, and all of the evaluation parameters are low. For the results of the CNN model, its recall rate reached 0.75, which is much better than that of the SPMG model. However, there is still some overidentification, leading to low precision, F1, and IoU. For the results of the Bin+CNN model, its recall rate is similar to that of the CNN model, and mainly because the overidentification of coffee flower is effectively reduced, the corresponding evaluation parameters (i.e., precision, F1, and IoU) are improved by 0.1, 0.11, and 0.08 compared with those of the CNN model, respectively. Therefore, for the small depression angle, the proposed method can improve the identification results of the coffee flower compared with the SPMG and CNN models.

#### 3.2.2. Results of the Image with a 52.5° Angle of Depression

The observed depression angle is increased to 52.5°, since the coffee flowers were not observed well under the 27.5° angle of depression. Therefore, the coffee flower image with a 52.5° angle of depression under soft lighting conditions is identified using the SPMG, CNN, and Bin+CNN models (Figure 9(c), Figure S4, and Table 4).

For the results of the SPMG model, the coffee flowers still cannot be well identified, and the corresponding recall rate and IoU are only 0.46 and 0.40, respectively. For the results of the CNN model, its recall rate reached 0.91, which is much better than that of the SPMG model. However, the corresponding precision is only 0.45, mainly because the boundary information of the coffee flower is enlarged. For the results of the Bin+CNN model, there is a clear advantage compared with the SPMG and CNN models, and the corresponding recall rate, precision, F1, and IoU have reached 0.85, 0.76, 0.80, and 0.67, respectively. In summary, the proposed method was able to achieve the ideal recall rate, and the overidentification of the coffee flower caused by the CNN model is noticeably reduced. Moreover, compared with the image with a 27.5°angle of depression, 52.5° is more conducive to coffee flower identification.

#### 3.2.3. Results of the Image with a 77.5° Angle of Depression

The shooting angle of coffee flowers is further increased to 77.5°, and the same identification process is carried out (Figure S5, Figure 9(d), and Table 5).

From the corresponding identification results, it can be seen that compared with the SPMG and CNN models, there are still similar advantages in the Bin+CNN model. For the results of the SPMG model, the recall rate reached 0.69, which is a clear improvement over the results with the other two depression angles, while it is still lower than that of the CNN and Bin+CNN models. For the results of the CNN model, its recall rate reached 0.96, and it can be seen that almost no other areas are misidentified except for the overidentification of the contours of the coffee flowers (Figure S5 (d)), which means that the CNN can effectively remove the background information left in the binarization results. Therefore, the Bin+CNN model is used to correct the boundary information of the results based on the CNN model. From the identification results of the Bin+CNN model, it can be seen that the overidentification of coffee flowers caused by the CNN model is effectively reduced (Figure 9(d)), and the precision of the Bin+CNN model has reached 0.72, which significantly improved, by 0.4, compared with the CNN model. Meanwhile, the advantages of the evaluation parameters corresponding to the Bin+CNN model are significantly higher than those of the SPMG model.

#### 3.2.4. Results of the Images under Intense Lighting Conditions

In order to further validate the robustness of the proposed method, coffee flower images with different depression angles (i.e., 27.5°and 52.5°) under intense lighting conditions are identified using different methods (i.e., SPMG, CNN, and Bin+CNN models) (Figures 9(e) and 9(f), Figure S6, Figure S7, and Table 6).

The advantages of the Bin+CNN model can be validated from the corresponding identification results. For the identification results based on the image with a 27.5° angle of depression, there is still a lot of missing recognition in the SPMG model; all of the corresponding evaluation parameters are the lowest, among which, recall rate is only 0.22. Compared with the SPMG model, the CNN can effectively improve the identification results, and the recall rate reached 0.68, which is 0.46 higher than that of the SPMG model, and other parameters, i.e., precision, F1, and IoU, are improved by 0.02, 0.15, and 0.16, respectively. As expected, on the basis of the CNN model, the identification results can be further optimized by the Bin+CNN model; its recall rate is similar to that of the CNN model; and precision, F1, and IoU reached 0.51, 0.57, and 0.40, respectively, a clear improvement compared with those of the SPMG and CNN models. Moreover, for the identification results based on the image with a 52.5° depression angle, each method has shown a significant improvement compared with those of the image with a 27.5° angle of depression. In summary, in terms of comprehensive evaluation, the Bin+CNN model can effectively improve the recognition results compared with the other two models. Consequently, it can be concluded that the Bin+CNN model can accurately identify coffee flower distribution with better robustness.

## 4. Discussion

Different from other small plants, the color of the coffee flower is white. Although there is a clear difference between a coffee flower and the background under soft lighting conditions, it is more likely to be confused with the background under strong illumination conditions. Moreover, different shooting angles also have an influence on coffee flower identification. Previous studies have focused on identifying green plants from background under a specific angle and illumination conditions using various color indexes [29, 32]. Therefore, in order to simply and effectively discriminate coffee flowers from a complex background, this paper adopts the binarization to optimize the overidentification of CNN to achieve accurate identification of white coffee flowers. From the acquired identification results, based on different shooting depression angles and illumination conditions, the proposed method has been proven to improve the coffee flower identification accuracy compared with that of the SPMG and CNN models.

For coffee flower images taken from the soft lighting conditions, when the depression angle is 27.5°, the recall rate of the Bin+CNN is 0.53 higher than that of the SPMG model, while it is similar to that of the CNN model. However, CNN and Bin+CNN have a lot of overidentification, since the coffee flower target obtained is not clear in the image with a 27.5° angle of depression. When the depression angle is increased to 52.5°, both of the identification results of the CNN and Bin+CNN models have a significant improvement compared to those of the depression angle of 27.5°, while the SPMG model still cannot recognize the coffee flower well. In particular, the recall rate of the CNN model reached 0.91, but with a low precision; the reason for this phenomenon is that boundary information of coffee flowers is enlarged, which causes much more overidentification. For the results of the Bin+CNN model, as we expected, it has effectively reduced the overidentification of the coffee flower compared with the results of the CNN model and achieved state-of-the-art performance, and the corresponding recall rate and precision reached 0.85 and 0.76, respectively. When the depression angle is further increased to 77.5°, the comprehensive evaluation parameters of the Bin+CNN model are still the best, and its recall rate, precision, F1, and IoU reached 0.83, 0.72, 0.77, and 0.63, respectively.

For the coffee flower images taken from the intense lighting conditions, when the depression angle of the image is 27.5°, identification results of the SPMG model are similar to those of images under the soft lighting conditions, which means that the coffee flowers cannot be recognized based on the SPMG using the images with a 27.5° angle of depression. As for the CNN models, compared with the SPMG model, its evaluation parameters (i.e., recall rate, precision, F1, and IoU) have a noticeable improvement and reached 0.68, 0.36, 0.47, and 0.31, respectively. The Bin+CNN model can further optimize the results of CNN, since the contour information of the coffee flower in the binarization result is retained, and its precision, F1, and IoU have been improved by 0.15, 0.10, and 0.09, respectively, compared with those of the CNN model. For the coffee flower image with a depression angle of 52.5°, the proposed method has similar advantages compared with the SPMG and CNN models. In summary, the proposed method can still effectively identify the coffee flowers in the images taken from intense lighting conditions.

## 5. Conclusion

This paper supposed that the binarization algorithm can optimize the boundary contour information extracted by the CNN model and verified it by digital images with coffee flowers. Based on this analysis, this paper innovatively combined the binarization algorithm and the CNN model for the first time to identify the coffee flowers in the original digital images. By comparing the identification results from various algorithms, we could conclude that the proposed method (i.e., Bin+CNN) has an excellent performance in coffee flower identification, the accuracies of images with a 52.5° angle of depression under soft lighting conditions are the highest, and the corresponding F1 and IoU have reached 0.80 and 0.67, respectively. Although clear advantages could be generated using the proposed method for coffee flower identification, operational coffee flower identification needs to solve the following issues. First, the method was proposed for a specific area, so the identification ability of the model would decline as the background information changes. This issue can be addressed by collecting more background information in different regions as the negative samples, and good prediction results can be achieved by the model trained using comprehensive training sets. Second, in the first step, the patch-based CNN inevitably caused overidentification of coffee flowers; this issue can be addressed by using a deep semantic segmentation model with high-quality training images, which will be studied in the future work. In general, the proposed method can provide a great foundation for further information extraction on anthesis detection, the relationship between flowering and fruiting, and other related applications.

## Figures and Tables

**Table 1:** The specific setting of the network structure.

Input	Output
Conv3 × 3 × 3 × 64	
Conv3 × 3 × 64 × 64	FC-2
MaxPooling	FC-4096
Conv3 × 3 × 64 × 128	FC-4096
Conv3 × 3 × 128 × 128	MaxPooling
MaxPooling	Conv3 × 3 × 512 × 512
Conv3 × 3 × 128 × 256	Conv3 × 3 × 512 × 512
Conv3 × 3 × 256 × 256	Conv3 × 3 × 512 × 512
Conv3 × 3 × 256 × 256	MaxPooling
MaxPooling	Conv3 × 3 × 256 × 512
Conv3 × 3 × 512 × 512	Conv3 × 3 × 512 × 512

**Table 2:** Evaluation parameters of the training image.

	Recall	Precision	F1	IoU
SPMG	0.73	0.81	0.77	0.63
CNN	0.93	0.42	0.58	0.41
Bin+CNN	0.89	0.73	0.80	0.67

**Table 3:** Evaluation parameters of image with a 27.5° angle of depression.

	Recall	Precision	F1	IoU
SPMG	0.19	0.44	0.27	0.16
CNN	0.75	0.17	0.28	0.16
Bin+CNN	0.72	0.27	0.39	0.24

**Table 4:** Evaluation parameters of image with a 52.5° angle of depression.

	Recall	Precision	F1	IoU
SPMG	0.46	0.77	0.56	0.40
CNN	0.91	0.45	0.60	0.43
Bin+CNN	0.85	0.76	0.80	0.67

**Table 5:** Evaluation parameters of image with a 77.5° angle of depression.

	Recall	Precision	F1	IoU
SPMG	0.69	0.74	0.71	0.56
CNN	0.96	0.32	0.48	0.32
Bin+CNN	0.83	0.72	0.77	0.63

**Table 6:** Evaluation parameters of images under intense lighting conditions.

Angle of depression	Method	Recall	Precision	F1	IoU
27.5°	SPMG	0.22	0.34	0.32	0.15
	CNN	0.68	0.36	0.47	0.31
	Bin+CNN	0.64	0.51	0.57	0.40
52.5°	SPMG	0.66	0.55	0.60	0.42
	CNN	0.83	0.34	0.48	0.32
	Bin+CNN	0.80	0.50	0.62	0.45

## Supplementary Materials

Supplementary Materials Supplementary Figure S1: the image acquisition device installed in the study area and images taken from different shooting angles. Supplementary Figure S2: coffee flower identification results of the training image based on the SPMG and CNN models. Supplementary Figures S3–S5: coffee flower identification results of images with depression angles of 27.5°, 52.5°, and 77.5° under soft lighting conditions based on the SPMG and CNN models. Supplementary Figures S6 and S7: coffee flower identification results of images with depression angles of 27.5° and 52.5° under intense lighting conditions based on SPMG and CNN models.
