# Supplementary material for: Coffee Flower Identification Using Binarization Algorithm Based on Convolutional Neural Network for Digital Images
Source: Plant Phenomics. 2020 Oct 6;2020:6323965. doi: 10.34133/2020/6323965 (PMC7706348; doi:10.34133/2020/6323965)

**Supplementary Materials**

**Figure S1.** The image acquisition device installed in the study area and images taken from different shooting angles. (a) Automatic agrometeorological observation apparatus in coffee plantation. (b) The 24 shooting angles, which are composed of 3 depression angles and 8 azimuth angles.


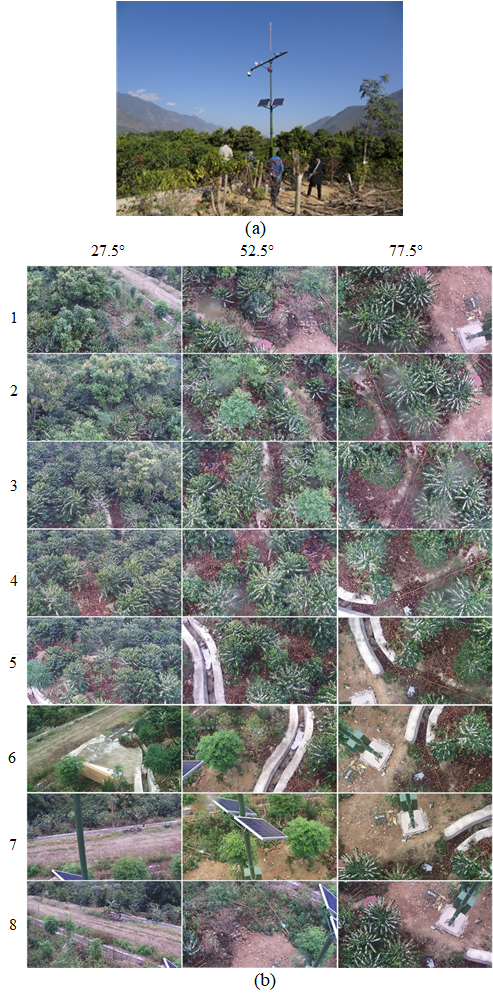


**Figure S2.** Coffee flower identification results of the training image based on the SPMG and CNN models. (a) Original input image. (b) Ground truth map. (c) and (d) are identification results of SPMG and CNN.


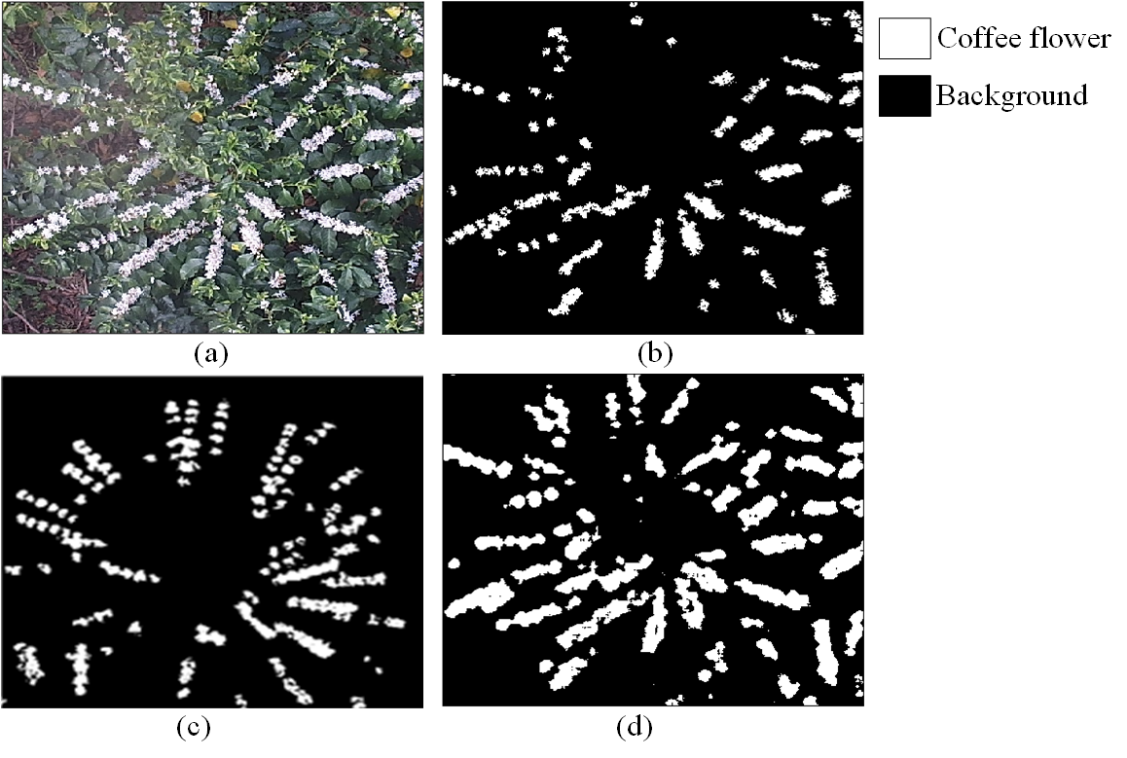


**Figure S3.** Coffee flower identification results of the image with depression angle of 27.5° under soft lighting conditions based on the SPMG and CNN models. (a) Original input image. (b) Ground truth map. (c) and (d) are identification results of the SPMG and CNN.


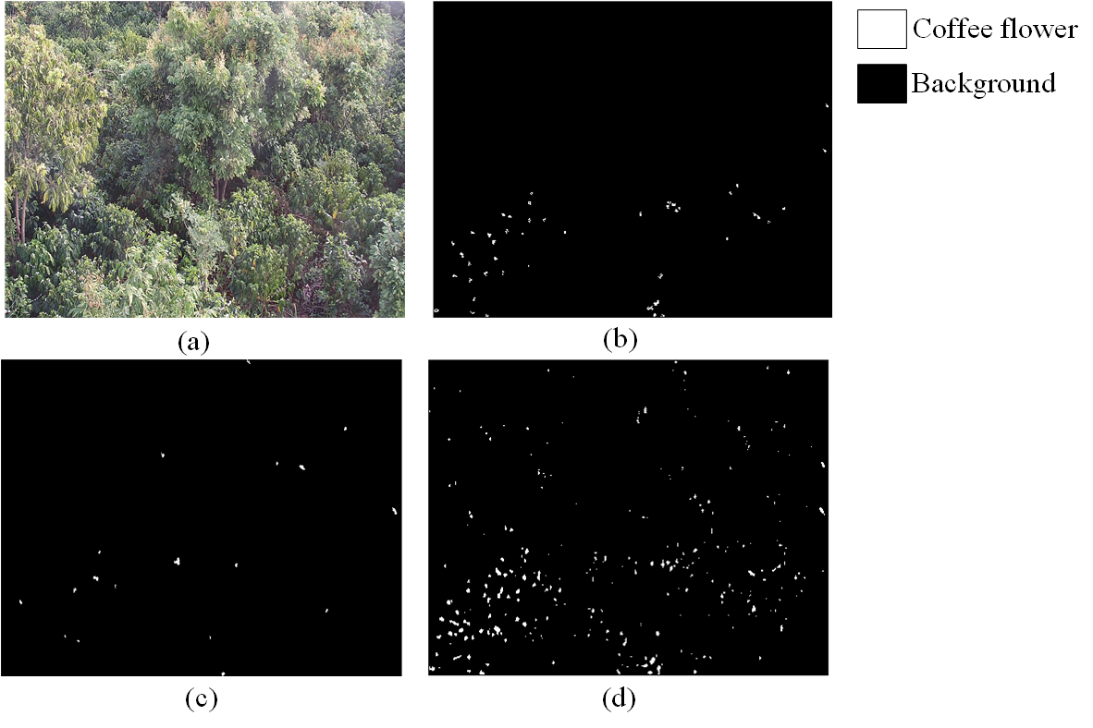


**Figure S4.** Coffee flower identification results of the image with depression angle of 52.5° under soft lighting conditions based on the SPMG and CNN models. (a) Original input image. (b) Ground truth map. (c) and (d) are identification results of SPMG and CNN.


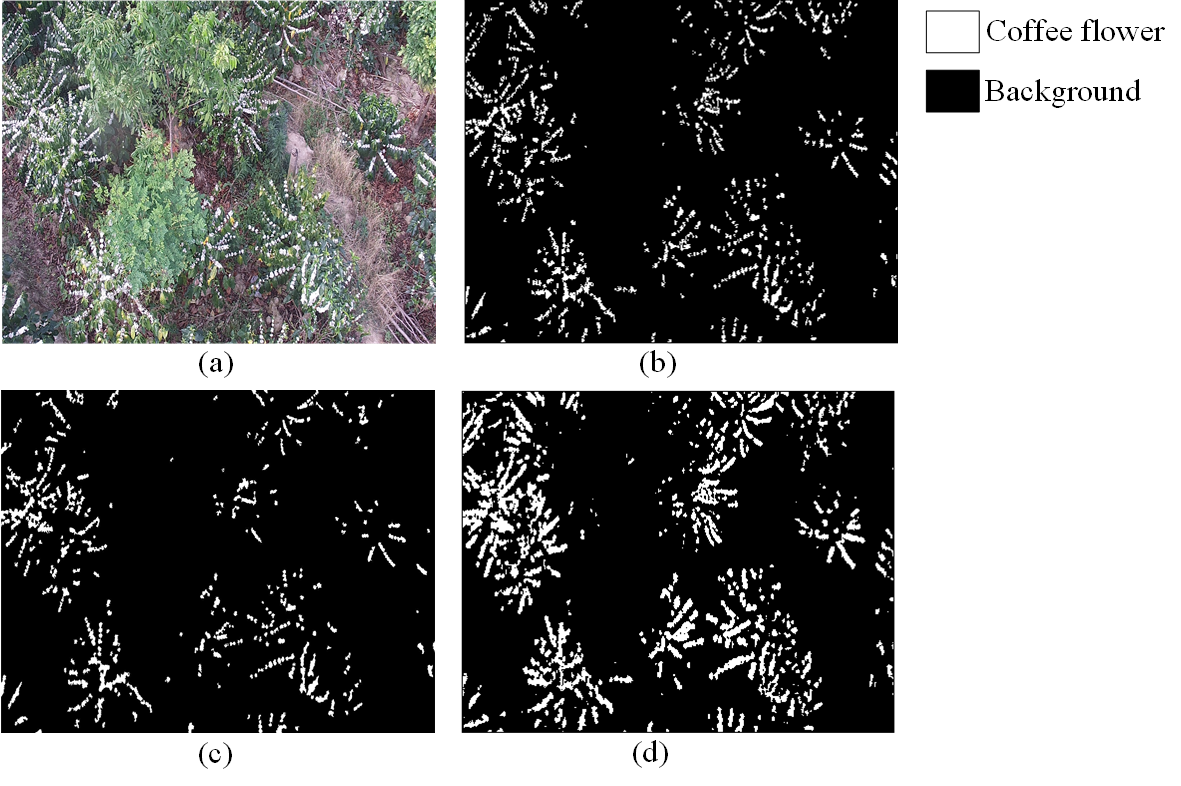


**Figure S5.** Coffee flower identification results of the image with depression angle of 77.5° under soft lighting conditions based on the SPMG and CNN models. (a) Original input image. (b) Ground truth map. (c) and (d) are identification results of SPMG and CNN.


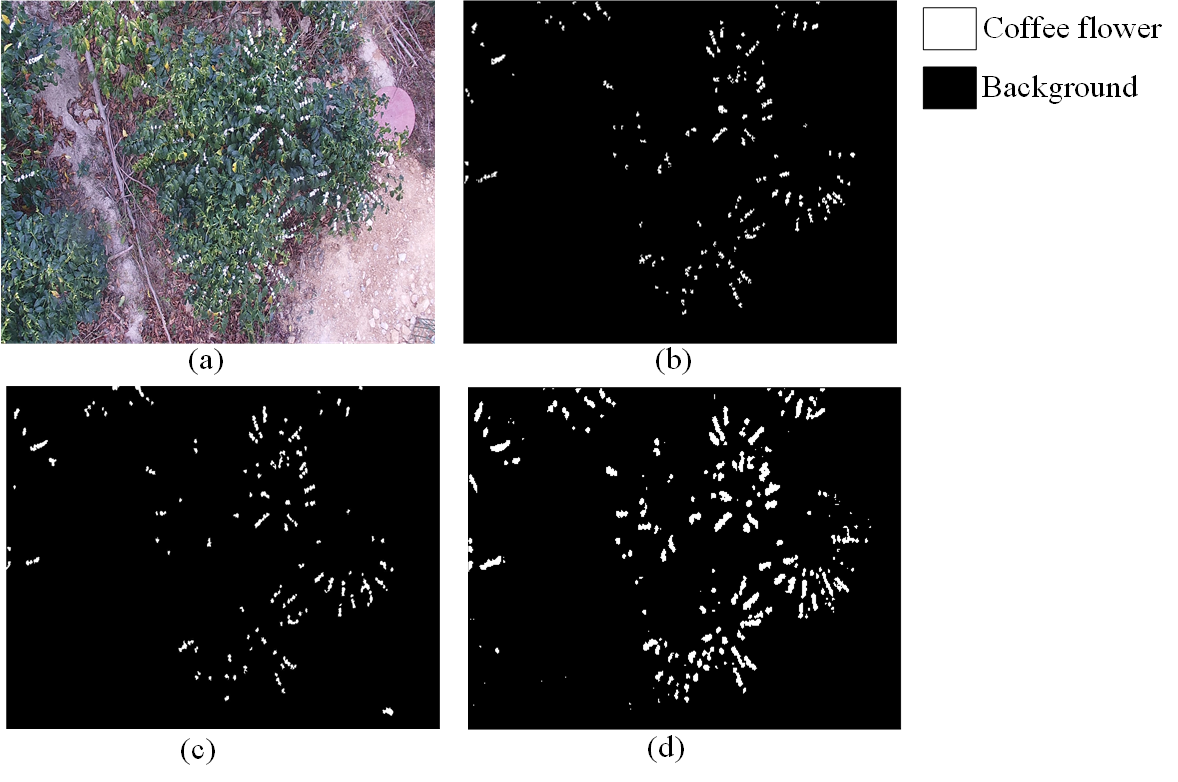


**Figure S6.** Coffee flower identification results of the image with depression angle of 27.5° under intense lighting conditions based on the SPMG and CNN models. (a) Original input image. (b) Ground truth map. (c) and (d) are identification results of SPMG and CNN.


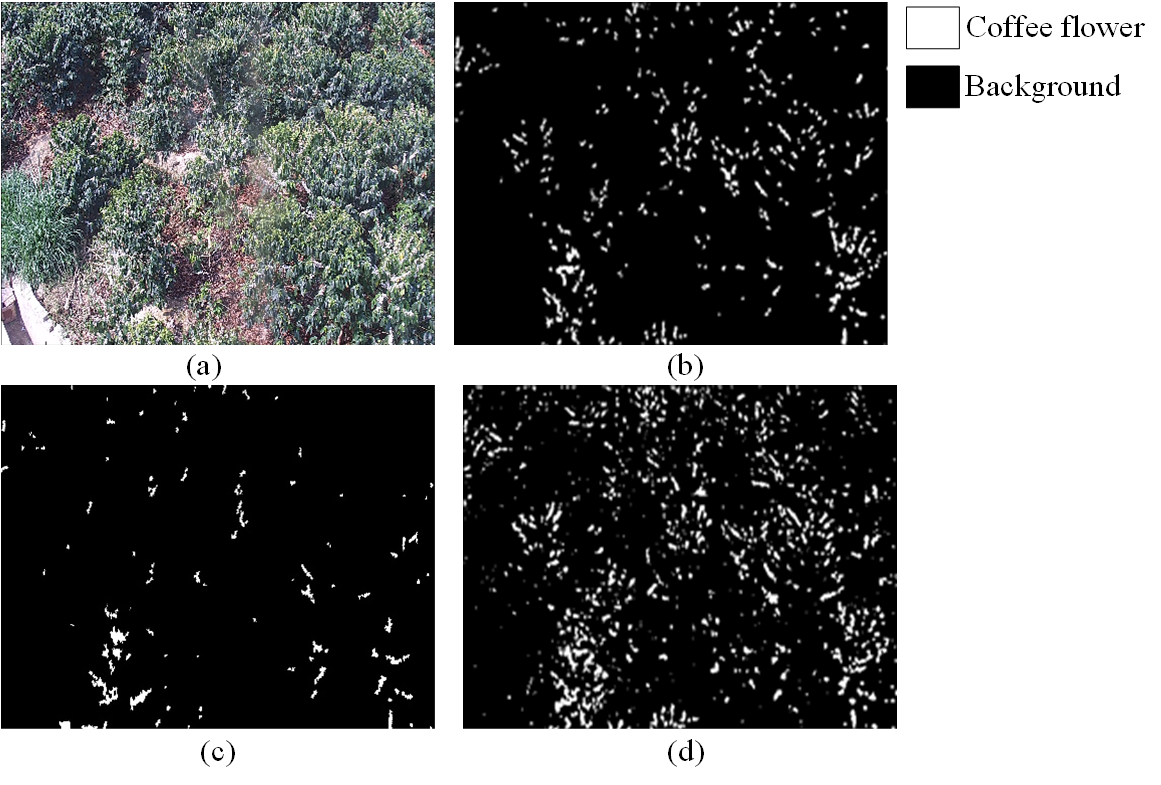


**Figure S7.** Coffee flower identification results of the image with depression angle of 52.5° under intense lighting conditions based on the SPMG and CNN models. (a) Original input image. (b) Ground truth map. (c) and (d) are identification results of SPMG and CNN.


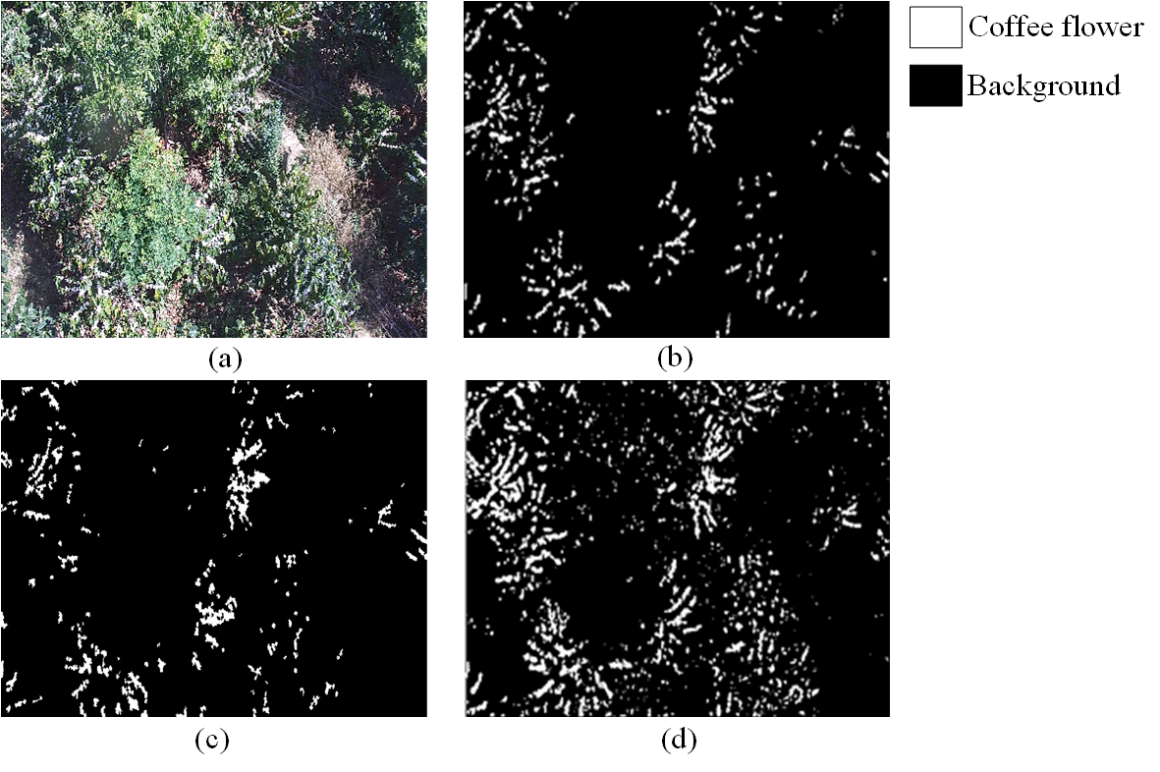

Supplement: Supplementary Materials — Supplementary Figure S1: the image acquisition device installed in the study area and images taken from different shooting angles. Supplementary Figure S2: coffee flower identification results of the training image based on the SPMG and CNN models. Supplementary Figures S3–S5: coffee flower identification results of images with depression angles of 27.5°, 52.5°, and 77.5° under soft lighting conditions based on the SPMG and CNN models. Supplementary Figures S6 and S7: coffee flower identification results of images with depression angles of 27.5° and 52.5° under intense lighting conditions based on SPMG and CNN models. [file 6323965.f1.docx]
